# Osteo-Sarcoma of the Femur—Recovery and Death

**Published:** 1893-09

**Authors:** C. A. White

**Affiliations:** Atlanta, Ga.


### OSTEO-SARCOMA OF THE FEMUR—RECOVERY AND DEATH.

BY C. A. WHITE, M. D., ATLANTA, GA.

When located at Cartersville, Ga., I was called to a hut, three miles from the city to see a young negro man, age 23 years, who had been suffering six months with a disease, believed by his people to be inflammatory rheumatism. By examination, his trouble was ascertained to be an osteo-sarcoma involving the whole length of the shaft of the femur. The size of the tumor was so great, that the patient could not lift it. Neither could others help him without giving pain and distress. His general appearance was that of one who had wasted away to skin and bone. His face was peaked and ashy. His eyes appeared dull, with a subdued look, varied occasionally by peculiar brilliancy seen in the hectic subject. His pulse was 144 and temperature 104, respectively. The tumor was about four times the size of the thigh. The sensation of crepitation very perceptible. The trabeculæ of bone, in breaking—under pressure—gave the sound of "crackling egg shells." The long muscles of the thigh were distended, separated and wasted almost to absence.

As there could be but one possible plan of procedure offering any satisfaction in the result, amputation, I gave him about forty hours to be prepared—saw him again in the meantime—and with my friends, Drs. Kirkpatrick and Calhoun, met the appointment. A long dinner table, a small deal table and a wash-pot full of boiling water, were furnished at the house. Every necessary for asepsis belonged to my armamentarium.

Wyeth's method, was followed, under aseptic principles learned from every source. A few words on the technique before the history of the recovery and death will be better, taken from *International Clinics*, Gaston April '91, which says: "The patient being placed in position, with the hip of the side to be operated on well over the corner of the table, the foot is elevated and an Esmarch bandage applied to drive the contained blood towards the heart. The bandage should not be tightly put over the seat of the disease for fear of driving septic matter into the circulation.

With the rubber bandage still in position, the needles are next introduced. Two steel matress needles, three-sixteenths of an inch in diameter and a foot long, are used. The point of one is inserted an inch and a half below the anterior superior spine of the ilium and slightly to the inner side of this prominence, and is made to traverse the muscles and deep fascia, passing about half way between the great trochanter and the iliac spine, external to the neck of the femur, and through the substance of the tensor vaginæ femoris, coming out just back of the trochanter. The point of the second needle is entered an inch below the level of the crotch internally to the saphenous opening, and passing through the abductors comes out about an inch and a half in front of the tuber-ischii. No vessels are endangered by these needles. The points are protected by corks to prevent injury to the operators hands.

A piece of strong white rubber tube, half an inch in diameter and long enough when tightened in position to go five or six times around the thigh, is now wound very tightly around and above the fixation needles and tied."

The method of amputation may be any that strikes the fancy of the surgeon. Mann's was adopted as to flaps. The bone sawn through and the heavy mass moved. Disarticulation then was an easy matter. Closing the wound, having a large drainage tube extending from acetabulum to the most dependent part, dry dressing, and removing to bed ended the operative procedure.

The record of the case is as follows:

W. M., colored, aged 23 years; Osteo-Sarcoma of the Left Femur. May 26, 1891, 3 p. m., pulse 144; Temperature 104. May 27, 3 p. m., pulse 144; Temperature 104. May 28, 10 a. m., Amputation at hip joint. May 28, 9 a. m., pulse 144; Temperature 104. May 29, 10 a. m., pulse 132; Temperature 103½. May 29, 9 p. m., pulse 132; Temperature 101 3-5. May 30, 4 p. m., pulse 132; Temperature 99 3-5. May 31, 4 p. m., pulse 132; Temperature 100 3-5. June 1, 4 p. m., pulse 120;

Temperature 99 3-5. June 2, 4 p. m., pulse 120 ; Temperature 99. June 3, 10 a. m., pulse 108; Temperature 98. June 6, p. m., pulse 102 ; Temperature 98 4-5. June 7, p. m., pulse 96 ; Temperature 98. June 11, p. m., pulse 96 ; Temperature 98 1-5. June 13, p. m., pulse 96 ; Temperature 98 1-5. June 19, p. m., pulse 96 ; Temperature 98. June 21, p. m., pulse 84 ; Temperature 98. June 23, p. m., pulse 90 ; Temperature 98.

This is the 27th day and the patient is in high spirits. Good appetite. Has gained about twenty pounds in weight. The first dressing was removed some ten days since, and wound redressed. Found healthy cicatrix almost fully cured. Allowed tubes to remain six days, redressed after gently withdrawing it. No pus, sound cicatrix, happy patient. Has had rocking chair and crutches several days. Recovery. Some six or seven days later I was called in haste to see him. It had been raining and he had slept in a draft. At 4 a. m., when I arrived he was threatened with pneumonia. Later, during the day, Dr. Kirkpatrick drove out with me. The disease developed and death followed in five days. My colleagues agree that the disease which killed him was independent of the surgical case.

